# Ldlr-/-.Leiden mice develop neurodegeneration, age-dependent astrogliosis and obesity-induced changes in microglia immunophenotype which are partly reversed by complement component 5 neutralizing antibody

**DOI:** 10.3389/fncel.2023.1205261

**Published:** 2023-06-29

**Authors:** Florine Seidel, Kees Fluiter, Robert Kleemann, Nicole Worms, Anita van Nieuwkoop, Martien P. M. Caspers, Nikolaos Grigoriadis, Amanda J. Kiliaan, Frank Baas, Iliana Michailidou, Martine C. Morrison

**Affiliations:** ^1^Department of Metabolic Health Research, Netherlands Organisation for Applied Scientific Research (TNO), Leiden, Netherlands; ^2^Department of Medical Imaging, Anatomy, Preclinical Imaging Center (PRIME), Radboud Alzheimer Center, Donders Institute for Brain, Cognition, and Behavior, Radboud University Medical Center, Nijmegen, Netherlands; ^3^Department of Clinical Genetics, Leiden University Medical Center, Leiden, Netherlands; ^4^Department of Microbiology and Systems Biology, Netherlands Organisation for Applied Scientific Research (TNO), Leiden, Netherlands; ^5^Laboratory of Experimental Neurology and Neuroimmunology and the Multiple Sclerosis Center, 2^nd^ Department of Neurology, AHEPA University Hospital, Aristotle University of Thessaloniki, Thessaloniki, Greece

**Keywords:** obesity, aging, brain, neurodegeneration, astrogliosis, neuroinflammation, anti-complement component 5

## Abstract

**Introduction:**

Obesity has been linked to vascular dysfunction, cognitive impairment and neurodegenerative diseases. However, experimental models that recapitulate brain pathology in relation to obesity and vascular dysfunction are still lacking.

**Methods:**

In this study we performed the histological and histochemical characterization of brains from Ldlr-/-.Leiden mice, an established model for obesity and associated vascular disease. First, HFD-fed 18 week-old and 50 week-old Ldlr-/-.Leiden male mice were compared with age-matched C57BL/6J mice. We then assessed the effect of high-fat diet (HFD)-induced obesity on brain pathology in Ldlr-/-.Leiden mice and tested whether a treatment with an anti-complement component 5 antibody, a terminal complement pathway inhibitor recently shown to reduce vascular disease, can attenuate neurodegeneration and neuroinflammation. Histological analyses were complemented with Next Generation Sequencing (NGS) analyses of the hippocampus to unravel molecular pathways underlying brain histopathology.

**Results:**

We show that chow-fed Ldlr-/-.Leiden mice have more severe neurodegeneration and show an age-dependent astrogliosis that is not observed in age-matched C57BL/6J controls. This was substantiated by pathway enrichment analysis using the NGS data which showed that oxidative phosphorylation, EIF2 signaling and mitochondrial dysfunction pathways, all associated with neurodegeneration, were significantly altered in the hippocampus of Ldlr-/-.Leiden mice compared with C57BL/6J controls. Obesity-inducing HFD-feeding did not aggravate neurodegeneration and astrogliosis in Ldlr-/-.Leiden mice. However, brains from HFD-fed Ldlr-/-.Leiden mice showed reduced IBA-1 immunoreactivity and increased CD68 immunoreactivity compared with chow-fed Ldlr-/-.Leiden mice, indicating alteration of microglial immunophenotype by HFD feeding. The systemic administration of an anti-C5 treatment partially restored the HFD effect on microglial immunophenotype. In addition, NGS data of hippocampi from Ldlr-/-.Leiden mice showed that HFD feeding affected multiple molecular pathways relative to chow-fed controls: HFD notably inactivated synaptogenesis and activated neuroinflammation pathways. The anti-C5 treatment restored the HFD-induced effect on molecular pathways to a large extent.

**Conclusion:**

This study shows that the Ldlr-/-.Leiden mouse model is suitable to study brain histopathology and associated biological processes in a context of obesity and provides evidence of the potential therapeutic value of anti-complement therapy against obesity-induced neuroinflammation.

## 1. Introduction

Obesity has become a major health burden with important social and economic impacts. It has been increasingly associated with various comorbidities including vascular dysfunction, cardiovascular abnormalities and atherosclerosis (Andolfi and Fisichella, 2018; Csige et al., 2018). Recently, obesity has been further linked to brain pathology and cognitive impairment (Tanaka et al., 2020). Several human studies showed that obesity is associated with brain abnormalities, including smaller total brain and grey matter volumes (Pannacciulli et al., 2006; Brooks et al., 2013) and a higher risk to develop dementia (Pedditizi et al., 2016). Excessive accumulation of fat in the context of obesity is known to trigger white adipose dysfunction and release of pro-inflammatory cytokines leading to chronic and systemic low-grade inflammation (Kiliaan et al., 2014). Systemic inflammation, in addition to obesity-related vascular dysfunction, can affect the integrity of the blood-brain barrier and promote neuroinflammation (García-García et al., 2022). However, the full mechanism underlying obesity-related brain impairment is still not fully understood. Research on biological processes involved in obesity-related brain impairment is notably limited by a lack of proper translational animal models for obesity. Up to date, most studies describing the effect of obesity on brain pathology involve animal models that either do not use diets comparable to those of humans (e.g., too high fat content), do not develop important phenotypical characteristics of human obesity such as insulin resistance and dyslipidemia, or lack extensive characterization (reviewed in Guillemot-Legris and Muccioli, 2017). Moreover, translational models reflecting a broader spectrum of obesity-associated comorbidities and neuropathology are still scarce.

The Ldlr-/-.Leiden mouse model is a preclinical model for obesity that recapitulates its associated comorbidities with established translational value (Morrison et al., 2018; van den Hoek et al., 2020). When fed an energy-dense high-fat diet (HFD; with a macronutrient composition that is comparable to that of human diets), Ldlr-/-.Leiden mice develop obesity, insulin resistance and dyslipidemia in addition to atherosclerosis, with the involvement of adipose tissue inflammation and increased gut permeability (Gart et al., 2021, 2022a; van den Hoek et al., 2021). Under HFD feeding, behavioural analyses and brain imaging have shown that Ldlr-.-/Leiden mice exhibit impaired spatial memory and reduced hippocampal volume (Arnoldussen et al., 2022). However, the underlying brain pathology on a histological and gene expression level in this mouse model is still not fully described.

On the cellular and molecular level, obesity has been shown to induce neurodegeneration, together with astrogliosis and neuroinflammation (Dorfman and Thaler, 2015). Astrocytes, the most abundant cells of the brain, are glial cells known to support neuronal function that also play essential roles in blood-brain barrier formation and maintenance, regulation of neuronal synaptogenesis and immune signaling (Giovannoni and Quintana, 2020). Following neuronal injury, astrocytes may become reactive, followed by proliferation and hypertrophy of their cell bodies and cytoplasmic processes (Eng and Ghirnikar, 1994). This process, known as astrogliosis, is characterized by an extensive synthesis of glial fibrillary acidic protein (GFAP) (Eng and Ghirnikar, 1994; Sofroniew, 2009). In both humans and rodents, obesity-induced astrogliosis was notably shown in the hypothalamus, as well as other parts of the brain such as the cortex and the hippocampus (Thaler et al., 2012; Guillemot-Legris and Muccioli, 2017), which is also accompanied by an increase in GFAP immunoreactivity (Guillemot-Legris and Muccioli, 2017; Bondan et al., 2019; Bandala et al., 2022). Obesity-related astrogliosis has been associated with neuroinflammation, which is characterized by microglia activation. As the immune cells of the brain, microglia can be activated upon stress stimuli and undergo phenotypical and morphological changes (Guillemot-Legris and Muccioli, 2017). Obesity has been shown to induce microglia activation in multiple areas of the brain, including the hypothalamus, cortex and hippocampus (Thaler et al., 2012; Dorfman and Thaler, 2015; Guillemot-Legris and Muccioli, 2017), which seems to be accompanied with changes in microglia immunophenotype: obesity-inducing HFD feeding in rodents has been shown to enhance the expression of the microglia-specific marker ionized calcium binding adapter molecule 1 (IBA-1) protein (Ito et al., 1998; Wahid et al., 2021) and to increase the number of IBA-1-positive microglia in the hippocampus (Thaler et al., 2012; Koga et al., 2014; Ahmad Tarmizi et al., 2022). However in humans, no difference in terms of IBA-1 density was observed in obese cases (Lier et al., 2019). Lier et al. further described the existence of areas exhibiting a loss of IBA-1 immunoreactivity while remaining immunopositive for other microglial markers. Consistent with this, a study suggested that HFD feeding in mice rather increases CD68-positive activated microglia in the hippocampus (Tucsek et al., 2014).

In parallel, it has been recently demonstrated that obesity increases the activity of the complement system in the brain, a part of the innate immune system implicated in host defence and inflammation (Graham et al., 2020). The complement system is activated through three major pathways, the classical, lectin and alternative pathways, all converging to the activation and cleavage of the downstream complement component C5 (Sarma and Ward, 2011). The activation of this terminal complement pathway notably results in the formation of the immunostimulating chemoattractant C5a and the terminal membrane attack complex (MAC). Activation of the complement system has been linked to systemic inflammation and atherosclerosis, two key components of obesity-related pathology (Vlaicu et al., 2016; Shim et al., 2020). In the brain, regulated complement system activation is essential for development as it mediates synaptic pruning (Stevens et al., 2007). However, in pathological conditions, overactivation of the complement system can also trigger neuroinflammatory cascades in which astrocytes and microglia are activated leading to the development of neurodegenerative diseases (Dalakas et al., 2020). Several studies further showed in acute neuroinflammatory conditions that the induction of neuroinflammation can be abrogated by inhibition of terminal complement system activation (Fluiter et al., 2014; Michailidou et al., 2018). However, in chronic neuroinflammation in a context of obesity, the implication of complement system activation and the therapeutic value of its inhibition are still poorly known.

In the present study we characterized the development of brain pathology in the Ldlr-/-.Leiden mouse model for obesity using (immuno)histology. To first understand the role of the Ldlr-/-.Leiden genotype, the development of brain histopathology was compared between young and aged Ldlr-/-.Leiden and aged-matched wild-type (C57BL/6J) mice. In parallel, the development of obesity-induced neuropathology was analysed in the Ldlr-/-.Leiden mice fed an obesity-inducing HFD compared with mice fed a standardized chow diet. We present data supporting that the genetically-determined impaired cholesterol metabolism is associated with brain neuroinflammation in this mouse model and further show that application of a HFD worsens the underlying brain pathology. To unravel potential underlying biological processes in the brain, gene expression was analysed in the hippocampus, the most important brain region involved in memory and learning. Finally, we tested in HFD-fed Ldlr-/-.Leiden mice whether an anti-complement therapy inhibiting systemic complement C5 (BB5.1 antibody; Zelek et al., 2020), shown to improve vascular function in the same mouse model (Seidel et al., 2022), can limit neurodegeneration and neuroinflammation. This study provides evidence supporting the Ldlr-/-.Leiden mouse model as a suitable model to study obesity-associated brain impairment against which anti-complement therapies may be promising.

## 2. Materials and methods

### 2.1. Animals

#### 2.1.1. Animals and housing

The studies were approved by an independent Animal Welfare Body (IVD TNO; approval numbers TNO-451 and TNO-499) under project licenses granted by the Netherlands Central Authority for Scientific Procedures on Animals (CCD; project license numbers AVD5010020172064 and AVD5010020172931). All animal experiments were performed in compliance with the European Union directive 2010/63/EU regarding the use of laboratory animals. Male C57BL/6J and Ldlr-/-.Leiden mice obtained from the breeding stock at TNO Metabolic Health Research (Leiden, the Netherlands) were group-housed (two to six animals per cage) in a conventional animal room (temperature ∼21°C, relative humidity 50–60%, light cycle 07:00 to 19:00) and received food and water *ad libitum*. Until the start of the studies, the animals were fed a standardized chow diet (Sniff R/M V1530, Uden, the Netherlands). Randomization, blinding methods and power calculations were used as previously described (Seidel et al., 2022).

#### 2.1.2. Effect of genetic background

**FIGURE 1:**
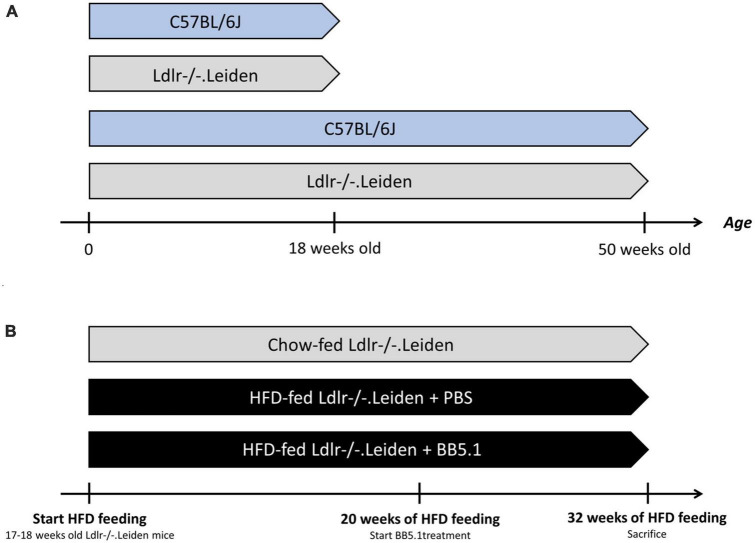
Experimental design. **(A)** To study the development of neuropathology over time, four groups of mice were fed a standardized chow diet: 18 week-old C57BL/6J mice (*n* = 8), 18 week-old Ldlr-/-.Leiden mice (*n* = 8), 50 week-old C57BL/6J mice (*n* = 8) and 50 week-old Ldlr-/-.Leiden mice (*n* = 8). **(B)** In a separate study, to investigate the effect of HFD feeding and an intervention on neuropathology development, two groups of Ldlr-/-.Leiden mice were fed an obesity-inducing HFD (*n* = 17 for the two groups). A separate group of chow-fed Ldlr-/-.Leiden mice served as a non-obese reference. During the last 20 weeks of HFD feeding, one group of HFD-fed groups received an BB5.1 antibody treatment while the other HFD-fed group and the chow-fed group received injections with PBS. The average age of the mice at the end the study was again 50 weeks.

To investigate the effect of genetic background on the development of neuropathology in Ldlr-/-.Leiden mouse model, two groups of Ldlr-/-.Leiden mice were terminated at 18 or 50 weeks of age (Figure 1A). For comparison, two groups of C57BL/6J mice were terminated at the same age. All groups were kept on chow diet. The mice were terminated by isoflurane inhalation (4%) and heart puncture followed by perfusion with phosphate-buffered saline (PBS) for 10 min (1 ml/min).

#### 2.1.3. Effect of HFD feeding and anti-complement C5 treatment

To investigate the effect of HFD feeding and treatment with an established antibody (BB5.1) targeting complement C5 on neuropathology in Ldlr-/-.Leiden mice, 17–18 week-old Ldlr-/-.Leiden mice were matched into two groups based on body weight, blood glucose, plasma cholesterol and plasma triglyceride levels (Figure 1B). These two groups were fed an energy-dense HFD [45 kcal% fat with 39 kcal% fat from lard and 6 kcal% fat from soybean oil, 20 kcal% protein and 35 kcal% carbohydrates, D12451, Research Diets, New Brunswick, NJ, USA, Gart et al., 2021] for 32 weeks. During the last 12 weeks of HFD feeding, one group of mice received intraperitoneal injections with an established anti-C5 antibody (BB5.1, 5 mg/mL in PBS, 1 mg/mouse; Zelek et al., 2020). The BB5.1 antibody was produced as reported (Seidel et al., 2022). A HFD-fed control group received intraperitoneal injections of PBS (as a vehicle control). The anti-C5 treatment or PBS was administered twice a week until the end of the study as we detailed previously (Seidel et al., 2022). Mice were euthanized at 50 weeks old using the aforementioned method. A separate group of chow-fed Ldlr-/-.Leiden mice received similar injections with PBS and was sacrificed at 50 weeks of age as a non-obese reference.

### 2.2. Brain histology

Brains were collected at sacrifice and a mid-sagittal cut was performed. The right hemisphere was fixed in phosphate-buffered formalin (3.7%) for one week. The samples were dehydrated over 2.5 days (Automatic Tissue Processor ASP300S, Leica Biosystems, Amsterdam, the Netherlands) and then embedded in paraffin blocks. 6 μm-thick sagittal cross-sections were cut and stained for further analyses. Neurodegeneration was assessed on cross-sections stained with hematoxylin-eosin (HE). For this analysis four anatomic locations were examined: cortex, the hippocampus, the thalamus and the hypothalamus. The grade for degeneration was based on the following rubric: (1) one of a few foci of degeneration; (2) up to 5% (estimated) of the cells or structures degenerated; (3) 5–15% (estimated) of the cells or structures degenerated; (4) 15–40% (estimated) of the cells or structures degenerated; (5): greater than 40% (estimated) of the cells or structures degenerated.

#### 2.2.1. Immunohistochemistry

Sagittal cross-sections were deparaffinized in xylene and rehydrated with alcohol gradients and demineralized water. The sections were stained for GFAP, IBA-1, CD68 and triggering receptor expressed on myeloid cells 2 (TREM2) as detailed in Table 1. Antigen retrieval was performed by heat in a water bath (96°C, 40 min) for GFAP and IBA-1 immunostaining or in citrate buffer (pH 6, 96°C, 20 min) using a Dako PT-link device (Dako, Glostrup, Denmark) for CD68 and TREM2 immunostaining.

**TABLE 1:** Immunohistochemical staining of brain cross-sections.

Antigen	Primary antibody	Secondary antibody
GFAP	Anti-GFAP, Z0334 (Dako), 1:500, 4°C, overnight	Biotinylated donkey anti-rabbit (Jackson Immunoresearch), 1:1,500, room temperature, 1 h
IBA-1	Anti-IBA-1, 019-19741 (Fujifilm), 1:1,000, 4°C, overnight	Biotinylated donkey anti-rabbit (Jackson Immunoresearch), 1:1,500, room temperature, 1 h
CD68	Anti-CD68, ab125212 (Abcam), 1:500, 4°C, overnight	Brightvision HRP (Immunologic), 1:1, room temperature, 1h
TREM2	Anti-TREM2, af1729 (R&D Systems), 1:400, 4°C, overnight	Biotinylated rabbit anti-sheep (Vector Laboratories), 1:500, room temperature, 1h

#### 2.2.2. Quantification of immunoreactivity

Quantification of immunoreactivity was performed on sections scanned with a Pathology Scanner Second Generation SG300 (Philips, Best, the Netherlands). For these analyses four anatomic locations were examined: the internal capsule, hippocampus, thalamus and hypothalamus. Non-overlapping images were acquired from the analysed groups from each of the aforementioned anatomic locations, at a 20× magnification for the CD68 and TREM2 immunostainings and at a 5 × magnification for the GFAP and IBA-1 immunostainings, using the Image Management System Viewer software (Philips). Quantitative analysis of immunostaining was performed using the ‘measurement’ function of the Image J software (Image Pro Plus 5.1, National Institutes of Health, Bethesda, USA). For each picture, the immunoreactive area was measured and divided by the total area of measurement. For measurement of the immunoreactive area a threshold was set and applied to all images (stained in a single batch). The percentage of immunoreactive area over the total area assessed was then calculated and plotted for each brain region. Average measurements per mouse were also calculated and plotted for each group.

### 2.3. Hippocampus gene expression and pathway analysis

The left hemispheres of the brains were snap frozen in liquid nitrogen. The hippocampi were isolated and used to prepare homogenates using glass beads and ribonucleic nucleic acid (RNA) was extracted as described (Salic et al., 2019). RNA integrity and concentration were examined for each sample using the RNA 6000 Nano LabChip kit and a bioanalyzer 2100 (both Agilent Technologies, Amstelveen, the Netherlands) and the samples were sequenced by GenomeScan BV (Leiden, the Netherlands). RNA sequencing and RNA counts processing were performed as reported previously (Gart et al., 2021; Seidel et al., 2022). Differentially expressed genes were determined using the Deseq2-pipeline (Love et al., 2014) with a statistical cut-off of *p*-value (*p*) < 0.05 and used for gene enrichment analysis across pathways and biological processes using the Ingenuity Pathway Analysis suite (IPA; www.ingenuity.com, accessed on 15 September 2022). The upstream regulator analysis tool of IPA was used to assess the activity of upstream regulators as detailed in Salic et al. (2019).

### 2.4. Analysis of chemokine and cytokine concentrations in brain homogenates

Homogenates of the cortex (∼80 mg tissue) and thalamus (∼25 mg tissue) were prepared in lysis buffer and subsequently analysed by multiplex analysis using a V-PLEX Custom Mouse Biomarkers set (Mesoscale discoveries [MSD], Maryland, USA) that includes ‘Proinflammatory Panel 1’ (IFN-γ, IL-1β, IL-2, IL-4, IL-6, KC/GRO (CXCL1), IL-10, TNF-α) and ‘Cytokine Panel 1’ (MCP-1, IL-33, IL-27-p28/IL-30, IL-17A/F and IP-10 for the cortex and MCP-1, IL-33, IL-17A/F, IP-10, IL-15, MIP-1α and MIP-2 for the thalamus) as described before (Gart et al., 2022b). Plates were read on a MESO QuickPlex SQ 120 reader (MSD). Protein concentrations were measured in the same homogenates using a BCA Protein Assay Kit (Thermo Fisher Scientific, Waltham, MA, USA) and cytokine levels were expressed per mg protein. IL-4 and INF-γ in the cortex and INF-γ, IL-2 and IL-4 in the thalamus were below the detection range and were not further considered in the results.

### 2.5. Statistical analyses

All statistics were performed with Prism (GraphPad software, v9, San Diego, CA, USA). The normality of the distributions were assessed using a Shapiro-Wilk test. Outliers were detected using the Grubbs test or the ROUT test (Q = 1%) and excluded from statistical analysis. When the distribution was normal, a one-way analysis of variance (ANOVA) was performed with a Bonferroni correction for multiple comparisons. When the data were not normally distributed, a non-parametric Mann-Whitney test or Kruskal-Wallis test were performed followed by a Dunn’s multiple comparison test to assess intergroup differences. The results were considered significant when *p* ≤ 0.05 (two-tailed) at a 95% confidence level. All data are expressed as mean ± standard deviation (SD).

## 3. Results

To investigate the development of neuropathology in Ldlr-/-.Leiden mouse model, brain histopathology and hippocampal gene expression were analysed in 18 and 50 week-old Ldlr-/-.Leiden mice fed a standardized chow diet. For comparison, age-matched C57BL/6J mice were included in these analyses.

### 3.1. Ldlr-/-.Leiden mice exhibit neurodegeneration and age-related astrogliosis

**FIGURE 2:**
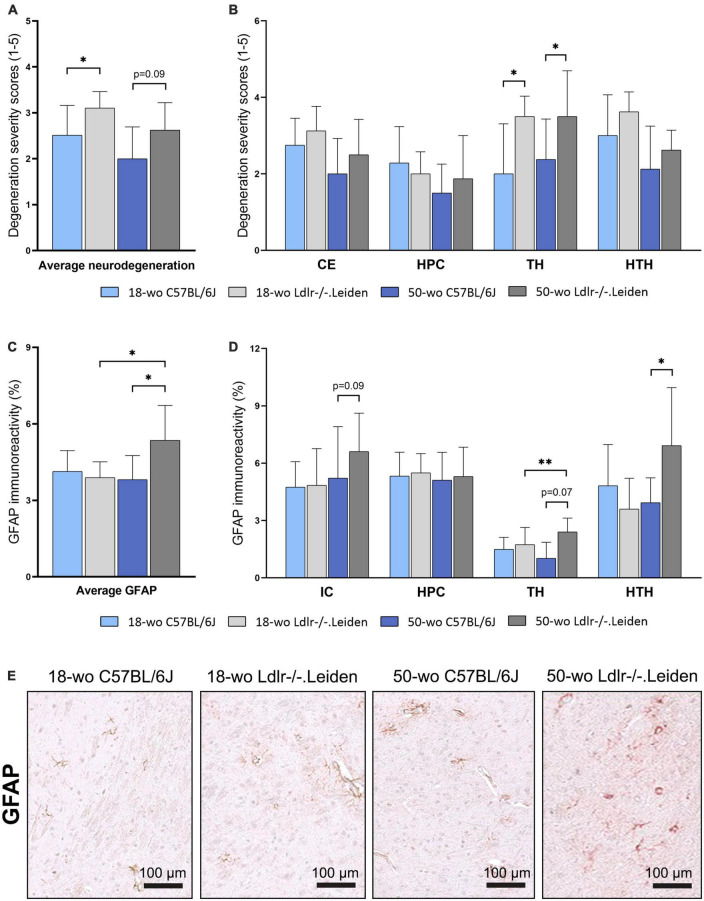
On chow diet, Ldlr-/-.Leiden mice developed neurodegeneration and age-dependent astrogliosis. **(A)** HE staining revealed that Ldlr-/-.Leiden mice developed more severe neurodegeneration than C57BL/6J mice and **(B)** the genotype effect was most pronounced in the thalamus. **(C)** Ldlr-/-.Leiden mice showed increased average GFAP immunoreactivity of all areas combined at 50 weeks of age, **(D)** which was mainly attributable to increases in the thalamus and hypothalamus. **(E)** Representative pictures of GFAP immunostaining in the brains of chow-fed C57BL/6J and Ldlr/-.Leiden mice of 18 weeks old and 50 weeks old respectively. *p ≤ 0.05, ^**^p ≤ 0.01. GFAP, glial fibrillary acidic protein; CE, cortex; HPC, hippocampus; TH, thalamus; HTH, hypothalamus; IC, internal capsule. Data are shown as mean ± SD.

Severity of degeneration, as assessed by a semi-quantitative scoring of HE-stained brain sections, was higher in chow-fed 18 week-old Ldlr-/-.Leiden mice than in the age-matched C57BL/6J mice (*p* = 0.034, Figure 2A), indicating an effect of the genotype on neurodegeneration in this model. The difference between the genotypes was most pronounced in the thalamus (*p* = 0.019, Figure 2B). The severity of degeneration in the thalamus (*p* = 0.037) and the average degeneration scores of all brain areas assessed (trends for significance, *p* = 0.090) remained higher in Ldlr-/-.Leiden mice than in C57BL/6J mice at 50 weeks of age. Quantification of GFAP immunoreactivity (astrogliosis) on consecutive slides, showed that the 18 week-old Ldlr-/-.Leiden and C57BL/6J mice exhibited similar GFAP immunoreactivity, whereas 50 week-old Ldlr-/-.Leiden mice showed increased GFAP immunoreactivity compared with C57BL/6J mice (*p* = 0.040, Figure 2C), especially in the hypothalamus (*p* = 0.040, Figure 2D) followed by the thalamus (trend for significance, *p* = 0.072) and internal capsule (trend for significance, *p* = 0.094). Representative pictures of GFAP immunostaining in chow-fed 18 and 50 week-old C57BL/6J and Ldlr-/-.Leiden respectively are provided in Figure 2E. In Ldlr-/-.Leiden mice, average GFAP immunoreactivity of all brain areas and GFAP immunoreactivity in the thalamus were significantly increased between 18 and 50 weeks of age (*p* = 0.038 and *p* = 0.007 respectively), suggesting that Ldlr-/-.Leiden mice develop an age-dependent astrogliosis that is not observed in C57BL/6J mice.

### 3.2. On gene expression level, Ldlr-/-.Leiden mice show an increase in mitochondrial dysfunction and a decrease in eiF2 signaling in the hippocampus

**FIGURE 3:**
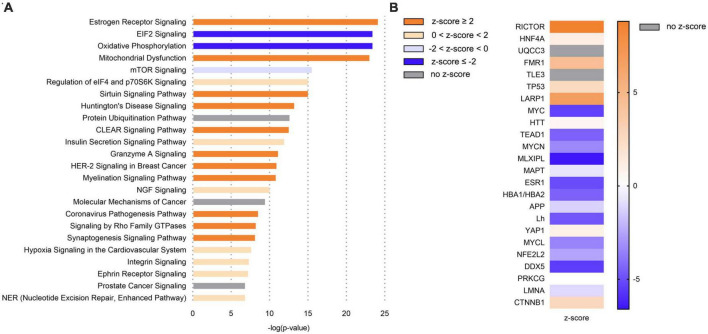
In comparison with C57BL/6J mice, Ldlr-/-.Leiden mice exhibited in the hippocampus a general overactivation of signaling pathways, including an increase in mitochondrial dysfunction, but also a downregulation of eiF2 signaling and oxidative phosphorylation. **(A)** Most significantly enriched canonical pathways based on hippocampal gene expression in chow-fed 50-wo Ldlr-/-.Leiden vs chow-fed 50-wo C57BL/6J mice. **(B)** Top 20 predicted upstream regulators (sorted by *p*-value) based on hippocampal gene expression in chow-fed 50-wo Ldlr-/-.Leiden vs chow-fed 50-wo C57BL/6J. The z-score indicates the predicted activation of a canonical pathway: z-score ≤ -2 indicates relevant inhibition of the pathway or regulator (indicated in dark blue); z-score ≥ 2 indicates relevant activation of the pathway or regulator (indicated in dark orange).

We next used transcriptomics analyses to characterize the molecular processes affected in the hippocampus, the main brain region involved in memory. Transcriptomics (NGS) followed by pathway enrichment analysis were performed in hippocampus mRNA of 50 week-old C57BL/6J and Ldlr-/-.Leiden mice. In comparison with C57BL/6J mice, Ldlr-/.Leiden animals showed alterations of many pathways in the hippocampus (∼200, Supplementary Table 1). The most enriched canonical pathways are displayed in Figure 3A. The pathways ‘Oxidative phosphorylation’ and ‘EIF2 signaling’ were the most inactivated and ‘Mitochondrial dysfunction’ was significantly increased. The subsequent upstream regulator analysis revealed that Rapamycin-insensitive companion of mammalian target of rapamycin (RICTOR) protein was the upstream regulator most activated while MLX-interacting protein-like (MLXIPL) was the most inactivated (Figure 3B). In addition, the ‘Synaptogenesis signaling pathway’ was activated and upstream regulators involved in protein synthesis [e.g., Fragile X Messenger Ribonucleoprotein 1 (FMR1), La Ribonucleoprotein 1 (LARP1)] were activated.

### 3.3. HFD feeding and anti-complement C5 treatment did not further alter neurodegeneration and astrogliosis in Ldlr-/-.Leiden mice

**FIGURE 4:**
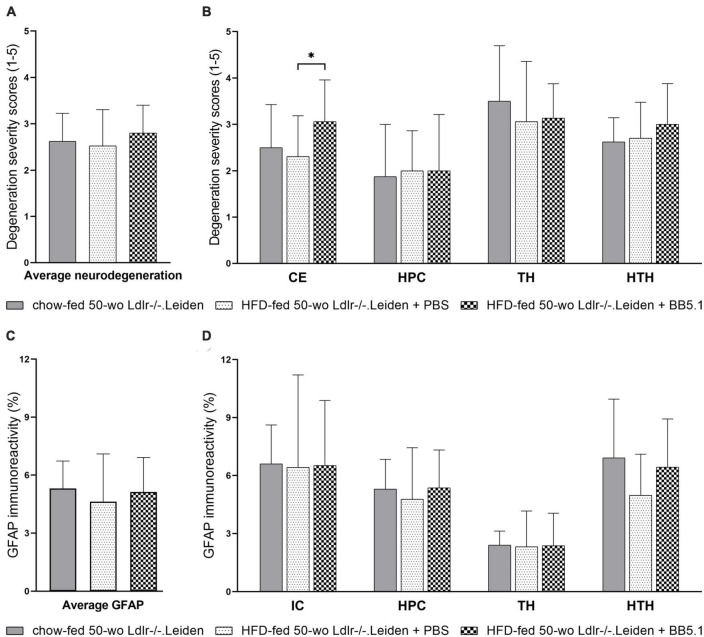
HFD feeding did not further aggravate neurodegeneration and gliosis in Ldlr-/-.Leiden mice. **(A)** HFD feeding did not affect average neurodegeneration of all areas combined or **(B)** neurodegeneration in individual brain regions. BB5.1 treatment had no effect except an increase in degeneration in the cortex. **(C,D)** HFD feeding and BB5.1 treatment did not alter GFAP immunoreactivity. *p ≤ 0.05. GFAP, glial fibrillary acidic protein; CE, cortex; HPC, hippocampus, TH, thalamus; HTH, hypothalamus; IC, internal capsule. Data are shown as mean ± SD.

Next, we investigated whether a subsequent addition of HFD feeding in Ldlr-/-.Leiden mice to induce obesity aggravates the metabolic disturbances in this model and potentially induces neuroinflammation. A separate group of Ldlr-/-.Leiden mice was fed an obesity-inducing HFD from 18 to 50 weeks of age and the aforementioned chow-fed group was used as a non-obese reference. We have previously shown that these HFD-fed Ldlr-/-.Leiden mice develop obesity and associated human-like dyslipidemia, NAFLD and atherosclerosis (Seidel et al., 2022). In the present study, HFD-fed 50 week-old Ldlr-/-.Leiden mice exhibited similar levels of neurodegeneration severity and similar amounts of GFAP immunoreactivity as the chow-fed animals in all anatomic areas examined (Figures 4A–D). To assess if an anti-complement therapy can rescue the brain health status, HFD-fed Ldlr-/-.Leiden mice were administered a systemic anti-complement C5 treatment (BB5.1 antibody) during the 12 last weeks of HFD feeding. BB5.1 treatment did not alter average neurodegeneration for all areas combined or neurodegeneration in the hippocampus, thalamus and hypothalamus. HFD-fed mice treated with BB5.1 treatment did however present with increased neurodegeneration in the cortex compared with HFD-fed control mice (*p* = 0.025). No effect of BB5.1 treatment was observed on GFAP immunoreactivity.

### 3.4. On HFD feeding, Ldlr-/-.Leiden mice exhibit changes in microglia immunophenotype, which were partially restored by an anti-complement C5 treatment

**FIGURE 5:**
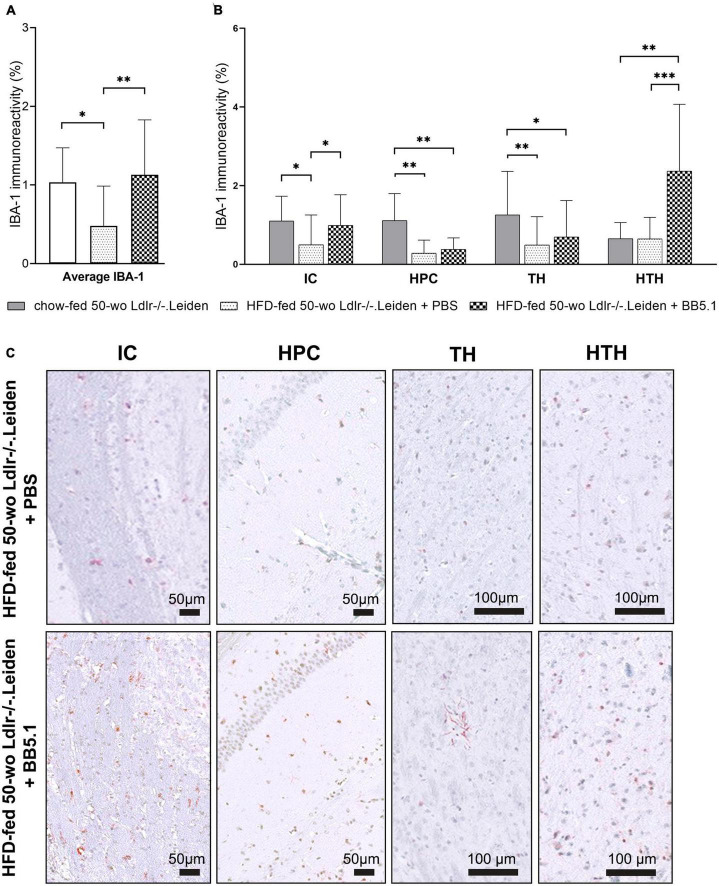
HFD feeding decreased IBA-1-positive microglia. **(A)** Average IBA-1 immunoreactivity was decreased by HFD feeding but restored with BB5.1 treatment, and **(B)** a pronounced reversal effect was observed in the internal capsule. **(C)** Representative pictures of IBA-1 immunostaining in individual brain regions of HFD-fed 50-wo Ldlr-/-.Leiden + PBS and HFD-fed 50-wo Ldlr-/-.Leiden + BB5.1 mice respectively. *p ≤ 0.05, ^**^p ≤ 0.01, ^***^p ≤ 0.001. IBA-1, Ionized calcium-binding adaptor molecule 1; IC, internal capsule; HPC, hippocampus; TH, thalamus; HTH, hypothalamus. Data are shown as mean ± SD.

**FIGURE 6:**
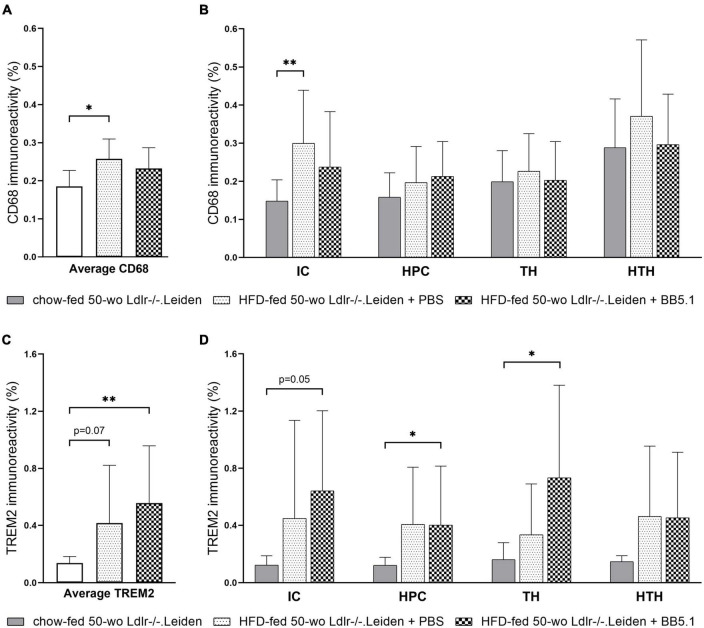
HFD feeding increased CD68-positive microglia and TREM2-positive microglia. **(A)** Average CD68 immunoreactivity was increased by HFD feeding but not altered by BB5.1 treatment. **(B)** HFD feeding specifically increased CD68 immunoreactivity in the internal capsule. **(C)** HFD feeding increased average TREM2 immunoreactivity (trend for significance) and BB5.1 treatment further increased TREM2 reactivity in comparison with chow-fed mice. **(D)** TREM2 immunoreactivity in individual brain regions was not altered by HFD feeding but was increased by BB5.1 treatment in comparison with chow-fed mice in the internal capsule, hippocampus and thalamus. *p ≤ 0.05, ^**^p ≤ 0.01. IC, internal capsule; HPC, hippocampus; TH, thalamus; HTH, hypothalamus. Data are shown as mean ± SD.

Although HFD feeding did not alter the development of neurodegeneration or astrogliosis, it did alter the microglia immunophenotype by decreasing the average IBA-1-positive immunoreactivity (*p* = 0.022, Figure 5A) while increasing the average of CD68-positive microglia (*p* = 0.010, Figure 6A). More specifically, analyses per anatomic region indicated that HFD-fed Ldlr-/-.Leiden mice showed lower IBA-1-positive immunoreactivity than chow-fed animals in the internal capsule (*p* = 0.013), hippocampus (*p* = 0.002) and thalamus (*p* = 0.006) but not in the hypothalamus (Figure 5B). BB5.1 treatment reversed the HFD-induced decrease in IBA-1 immunoreactivity: HFD-fed mice treated with BB5.1 exhibited increased average IBA-1 immunoreactivity relative to HFD-fed control mice (*p* = 0.008) which was notably attributable to increased IBA-1 immunoreactivity in the internal capsule (*p* = 0.015). BB5.1 treatment did not affect IBA-1 immunoreactivity in the hippocampus or thalamus. In the hypothalamus, HFD-fed mice treated with BB5.1 showed more IBA-1-positive microglia compared with HFD controls (*p* = 0.001) and chow-fed mice (*p* = 0.006). Representative pictures of IBA-1 immunostaining are displayed in Figure 5C.

To study whether the observed reduction in IBA-1 immunoreactivity induced by HFD feeding was due to microglial cell depletion or the result of a shift of the microglial cell immunophenotype, we examined the presence of CD68-positive and TREM2-positive microglial cells. HFD-fed Ldlr-/-.Leiden mice exhibited more CD68 immunoreactivity than chow-fed mice (*p* = 0.010, Figure 6A) which was attributable to a difference in the internal capsule specifically (*p* = 0.002, Figure 6B). No difference in CD68 immunoreactivity in the hippocampus, thalamus or hypothalamus was observed. BB5.1 treatment did not affect average CD68 immunoreactivity or CD68 immunoreactivity in the individual brain regions investigated. In addition, HFD-fed mice presented increased average TREM2-positive microglia compared to chow-fed mice (trend for significance, *p* = 0.069 Figure 6C) and HFD-fed mice treated with BB5.1 showed similar average TREM2 immunoreactivity as HFD controls. While no HFD feeding effect was observed in the individual brain regions, HFD-fed mice treated with BB5.1 showed higher average TREM2 immunoreactivity than chow-fed mice (*p* = 0.007) and higher TREM2 immunoreactivity in the internal capsule (trend for significance, *p* = 0.052), hippocampus and thalamus (*p* = 0.017 and *p* = 0.023 respectively, Figure 6D).

### 3.5. HFD feeding in Ldlr-/-.Leiden mice upregulates ‘neuroinflammation signaling’ and downregulates ‘synaptogenesis signaling’ pathways in the hippocampus while the anti-complement C5 treatment partially reverses hippocampal gene expression

**FIGURE 7:**
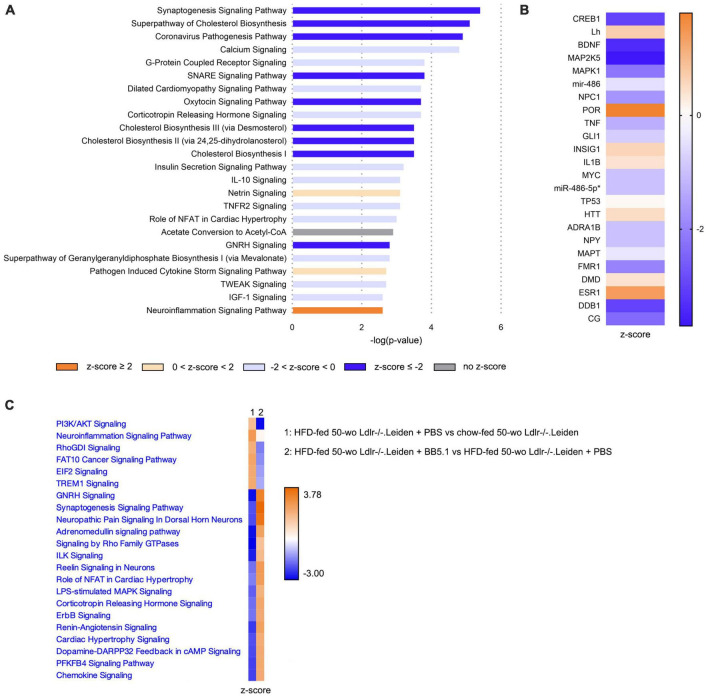
In Ldlr-/-.Leiden mice hippocampus, HFD feeding overall downregulated signaling pathways and increased neuroinflammation, while BB5.1 partly restored hippocampal gene expression. **(A)** Most significantly enriched canonical pathways based on hippocampal gene expression in HFD-fed 50-wo Ldlr-/-.Leiden + PBS vs chow-fed 50-wo Ldlr-/-.Leiden mice. **(B)** Top 20 predicted upstream regulators (sorted by *p*-value) based on hippocampal gene expression in HFD-fed 50-wo Ldlr-/-.Leiden vs chow-fed 50-wo Ldlr-/-.Leiden mice. **(C)** Comparison between canonical pathways significantly enriched in HFD-fed 50-wo Ldlr-/-.Leiden vs chow-fed 50-wo Ldlr-/-.Leiden mice and canonical pathways significantly enriched in HFD-fed 50-wo Ldlr-/-.Leiden + BB5.1 vs HFD-fed 50-wo Ldlr-/-.Leiden + PBS mice. The z-score indicates the predicted activation of a canonical pathway: z-score ≤ -2 indicates relevant inhibition of the pathway or regulator (indicated in dark blue); z-score ≥ 2 indicates relevant activation of the pathway or regulator (indicated in dark orange).

Transcriptomics analyses in the hippocampus further revealed that HFD significantly inactivated the ‘Synaptogenesis signaling’ and ‘SNARE signaling’ pathways (Figure 7A). A full list of the canonical pathways significantly enriched by HFD feeding is provided in Supplementary Table 2. Consistently, the upstream regulator brain-derived neurotrophic factor (BDNF) was significantly inactivated by HFD feeding (Figure 7B). Compared with the chow-fed mice, HFD-fed mice also exhibited significant inhibition of signaling pathways involved in cholesterol biosynthesis (e.g., ‘Superpathway of cholesterol biosynthesis’, ‘Cholesterol biosynthesis II’ and ‘Cholesterol biosynthesis III’). In addition, HFD feeding significantly activated the ‘Neuroinflammation signaling’ pathway, while enriching signalling downstream from interleukin 1β (IL1B, z-score 0.4) and tumour necrosis factor (TNF, z-score -1.3). BB5.1 treatment partially restored HFD-induced changes in hippocampal gene expression (Figure 7C): while HFD feeding inactivated ‘Synaptogenesis signaling’, BB5.1 reversely activated the pathway. Although BB5.1 treatment did not affect ‘Neuroinflammation signaling’, it tended to revert the HFD-induced inhibition of ‘Chemokine signaling’ and overactivation of ‘triggering receptor expressed on myeloid cells 1 (TREM1) signaling’. The complete list of the canonical pathways that were significantly enriched by BB5.1 treatment is provided in Supplementary Table 3.

### 3.6. HFD feeding increases concentrations of IL-6 in the cortex and KC in the thalamus which are not altered by anti-complement C5 treatment

To further examine the effect of HFD feeding and BB5.1 treatment on neuroinflammation in Ldlr-/-.Leiden mice, cytokines and chemokines were measured in cortex and thalamus homogenates. In the cortex, HFD feeding specifically increased IL-6 concentrations (*p* = 0.017) and BB5.1 treatment did not alter this effect (Table 2). HFD-fed Ldlr-/-.Leiden mice treated with BB5.1 showed higher cortical concentrations of interleukin 33 (IL-33, *p* = 0.013), interleukin 1β (IL-1β, *p* = 0.030), tumor necrosis factor (TNF-α, *p* = 0.008) and interleukin 10 (IL-10, trend for significance, *p* = 0.052) compared to chow-fed animals. However, no difference in these concentrations were observed between HFD-fed and chow-fed mice or between HFD-fed animals treated with BB5.1 and HFD-fed controls. In the thalamus, HFD feeding increased the concentration of KC, the mouse homologue of the human growth-regulated oncogene (GRO) chemokine (trend for significance, *p* = 0.057, Table 3). BB5.1 treatment did not alter KC concentrations but HFD-fed mice treated with BB5.1 exhibited higher IL-33 concentrations compared with chow-fed mice (*p* = 0.012) and HFD-fed controls (*p* = 0.031).

**TABLE 2:** Effect of HFD feeding and BB5.1 treatment on chemokines and cytokines concentrations in cortex homogenates of 50 week-old Ldlr-/-Leiden mice.

Cytokines	Chow		HFD + PBS		HFD + BB5.1	
	Mean	SD	Mean	SD	Mean	SD
IL-17A/F	0.13	0.05	0.14	0.06	0.15	0.05
IL-27p28/IL-30	0.28	0.15	0.26	0.12	0.28	0.10
**IL-33**	**38.82^a^**	**31.75**	**78.50 ^a.b^**	**56.42**	**96.85^b^**	**45.84**
IP-10	0.79	0.11	0.89	0.31	0.86	0.22
MCP-1	1.94	0.58	1.89	0.35	2.16	0.49
IL-10	0.09^a^	0.05	0.14^a.b^	0.05	0.15^b^	0.04
**IL-1β**	**0.09^a^**	**0.06**	**0.13^a.b^**	**0.07**	**0.16^b^**	**0.07**
IL-2	0.03	0.01	0.03	0.01	0.03	0.01
**IL-6**	**0.29^a^**	**0.16**	**0.41^b^**	**0.07**	**0.44^b^**	**0.06**
KC/GRO	0.96	0.30	0.86	0.13	0.99	0.14
**TNF-α**	**0.02^a^**	**0.01**	**0.03^a.b^**	**0.01**	**0.04^b^**	**0.01**

Concentrations are expressed a pg/mg protein. Cytokines that differed between groups are marked in bold (*p* ≤ 0.05) or underlined (trend for significance, *p* ≤ 0.1). For these cytokines, groups with corresponding superscript letters are statistically comparable (*p* > 0.05).

**TABLE 3:** Effect of HFD feeding and BB5.1 treatment on chemokines and cytokines concentrations in thalamus homogenates.

Cytokines	Chow		HFD + PBS		HFD + BB5.1	
	Mean	SD	Mean	SD	Mean	SD
IL-15	6.08	4.07	6.53	4.87	7.09	4.21
IL-17A/F	0.52	0.40	0.36	0.26	0.53	0.30
**IL-33**	**295.96^a^**	**209.64**	**375.97^a^**	**224.7**	**568.55^b^**	**190.01**
IP-10	3.48	1.21	3.81	1.51	3.25	0.99
MCP-1	2.07	0.59	2.40	0.57	2.40	0.45
MIP-1α	1.96	0.76	2.19	1.02	2.76	1.23
MIP-2	0.35	0.11	0.31	0.06	0.41	0.11
IL-10	0.36	0.20	0.35	0.32	0.36	0.21
IL-1β	0.04	0.02	0.06	0.05	0.06	0.04
IL-6	0.90	0.60	0.79	0.46	0.84	0.35
KC/GRO	1.52^a^	0.31	1.90^b^	0.45	1.92^b^	0.26
TNF-α	0.03	0.02	0.04	0.03	0.04	0.02

Concentrations are expressed a pg/mg protein. Cytokines that differed between groups are marked in bold (*p* ≤ 0.05) or underlined (trend for significance, *p* ≤ 0.1). For these cytokines, groups with corresponding superscript letters are statistically comparable (*p* > 0.05).

## 4. Discussion

Using (immuno)histological and hippocampal gene expression analyses, we showed that the Ldlr-/-.Leiden mouse model, an established translational model for obesity and related comorbidities, presents key signs of neurodegeneration and neuroinflammation, the severity of which is aggravated by aging and HFD feeding. In comparison with C57BL/6J mice, Ldlr-/-.Leiden mice exhibited more severe neurodegeneration and an age-dependent astrogliosis, especially in the thalamus. Transcriptomics analyses of RNA obtained from the hippocampus, the most important region of the brain involved in memory and cognition, further showed that Ldlr-/-.Leiden mice exhibited impaired oxidative phosphorylation and protein synthesis and repair (eiF2 signaling), in combination with increased mitochondrial dysfunction already on a chow diet. Application of obesity-inducing HFD feeding in Ldlr-/-.Leiden mice further triggered changes in microglia immunophenotype: HFD feeding reduced the reactivity of the IBA-1 marker for microglial cells and increased the CD68 immunoreactivity and TREM2 immunoreactivity (trend). HFD-induced neuroinflammation was accompanied by an increase in the protein concentration of IL-6 in the cortex and KC in the thalamus. On the gene expression level, HFD feeding increased neuroinflammation while inactivating the synaptogenesis signaling pathway. We further showed that this neuroinflammation can be modulated therapeutically: HFD-fed Ldlr-/-.Leiden mice responded to a therapeutic antibody intervention targeting complement C5 (BB5.1 antibody) which was previously shown to decrease neuroinflammation in acute models of neurodegenerative disease (Fluiter et al., 2014; Michailidou et al., 2018) and atherosclerosis in this mouse model (Seidel et al., 2022). The antibody treatment partially reverted the HFD-induced changes in microglial immunophenotype by increasing IBA-1 immunoreactivity without affecting CD68 and TREM2 immunoreactivities or cytokine levels in the brain. Hippocampal gene expression was also mostly reverted by the antibody treatment: the anti-C5 treatment notably reverted the HFD-induced inactivation of the synaptogenesis pathway; however, without affecting the neuroinflammation pathway.

Histopathological analyses of brain cross-sections showed that, already on a chow diet, Ldlr-/-.Leiden mice show signs of neurodegeneration as well as an aging-dependent astrogliosis that is not observed in C57BL6/J mice. When Ldlr-/-.Leiden mice were fed an obesity-inducing HFD, these pathological features remained. Neurodegeneration and astrogliosis are key features of obesity-related brain histopathology as described in both humans and mice (Thaler et al., 2012; Guillemot-Legris and Muccioli, 2017; Bondan et al., 2019; Bandala et al., 2022). In this study, neurodegeneration was prominently observed in the thalamus. Although neurodegeneration in the thalamus is poorly described in other rodent models, several human studies showed a reduction in grey matter volume in the thalamus in obese subjects compared to lean subjects (reviewed in Gómez-Apo et al., 2021), suggesting the development of an obesity-associated degeneration of the thalamus. In addition, the thalamus has been described to be sensitive to the development of lacunes related to cerebral small vessel disease that correlate with subsequent cognitive impairment (Benisty et al., 2009). At older age (50 weeks old), Ldlr-/-.Leiden mice also exhibited increased astrogliosis in the hypothalamus compared to C57BL6/J mice, which is consistent with the obesity-induced hypothalamic injury extensively described in literature (Thaler et al., 2012; Guillemot-Legris and Muccioli, 2017).

Neurodegeneration and astrogliosis in the context of obesity have been tightly linked to neuroinflammation (Dorfman and Thaler, 2015). In this study, HFD feeding induced changes in marker expression on microglial cells. We especially observed a reduction of IBA-1-positive microglia which was most pronounced in the internal capsule, hippocampus and thalamus. In animal models obesity-induced neuroinflammation has been mostly associated with an increase in IBA-1 immunoreactivity (Thaler et al., 2012; Koga et al., 2014; Ahmad Tarmizi et al., 2022). Depending on the age and duration of HFD feeding a similar increase in IBA-1 immunoreactivity has also been observed previously in Ldlr-/-.Leiden mice (Arnoldussen et al., 2017, 2022). In humans however, a recent study showed no differences in IBA-1 density between lean and obese subjects (Lier et al., 2019). The latter study also showed the existence of areas exhibiting a loss of IBA-1 immunoreactivity in the brain, areas that seemed related to the hepatic dysfunction of the patients (which is a comorbidity of obesity) rather than obesity itself. In line with this, Ldlr-/-.Leiden mice fed a HFD have been shown to develop hepatic dysfunction (van den Hoek et al., 2020; Gart et al., 2023) and the findings presented herein indicate that obese Ldlr-/-.Leiden mice also resemble human obesity-related brain pathology.

While HFD feeding decreased IBA-1 immunoreactivity, it increased CD68 immunoreactivity, especially in the white matter areas. Consistent with this, a previous study on C57BL/6 mice showed an increase in phagocytic CD68-positive microglia upon HFD feeding (Tucsek et al., 2014). A post-mortem study on elderly people further described the existence of microglia that are positive for CD68 but negative for IBA-1, which were found to be increased in deep subcortical white matter lesions (areas of abnormal myelination) (Waller et al., 2019). Interestingly, post-mortem analyses of the middle temporal gyrus in Alzheimer’s disease demonstrated that the state of dementia was positively associated with CD68 microglia marker expression while negatively correlating with IBA-1 (Minett et al., 2016). Inversely, the same study showed that in people without dementia, cognitive function was positively correlated with IBA-1 but negatively with CD68. The increase in CD68-positive microglia suggests that HFD-induced obesity promotes microglia phagocytic activity (Lier et al., 2021). In addition, in this study we observed a tendency towards an increase in TREM2 immunoreactivity after HFD feeding which further supports that HFD feeding drives phagocytic activity (Neumann and Takahashi, 2007) in the microglial cells in the Ldlr-/-.Leiden model. TREM2, one of the most highly expressed receptors on microglia, is regarded as an important player in the transition of microglia from homeostatic to pathological state in the development of Alzheimer’s disease (Qin et al., 2021). Altogether, this data suggests that HFD feeding induces a shift in microglia immunophenotype. A more extensive characterization of the expression of markers on microglial cells may further substantiate the microglia phagocytic activity and provide information on the microglia activation state.

To study potential molecular mechanisms underlying brain pathology in Ldlr-/-.Leiden mice, gene expression data were analyzed in the hippocampus, the brain structure involved in memory and learning. Compared with 50 week-old C57BL/6J mice, Ldlr-/-.Leiden of the same age showed a inactivation of oxidative phosphorylation and an increase in mitochondrial dysfunction, in conjunction with a strong downregulation of the elongation factor eIF2 signaling pathway. The eiF2 signaling pathway is critical for mRNA translation in protein synthesis and has been shown to be important for cellular repair and replacement of dysfunctional cells or organelles, and for long-term synaptic plasticity and memory (Sutton and Schuman, 2006; Rios-Fuller et al., 2020). These features were already observed in Ldlr-/-.Leiden mice on a chow diet indicating that the Ldlr-/-.Leiden model as such (i.e., without HFD feeding) replicates human hallmarks of brain pathophysiology that cannot be studied in aged wildtype C57BL/6J mice, namely impaired mitochondrial function and protein synthesis (Cui et al., 2012; Anisimova et al., 2018). Mitochondrial dysfunction is one of the central mechanisms that can lead to an energy crisis in brain cells and has been proposed as a determinant feature in neurodegeneration and the development of neurodegenerative diseases (Mattson et al., 2008; Belenguer et al., 2019). For instance, pharmacological inhibition of mitochondrial function in the brain has been shown to increase the permeability of the blood-brain barrier *in vivo* and *in vitro* (Doll et al., 2015), and mitochondrial dysfunction in astrocytes has been suggested to impact energy supply of neurons (Cunnane et al., 2021). Furthermore, while physiological concentrations of reactive oxygen species fulfil a signalling role, their overproduction is detrimental and is associated with lipid peroxidation and DNA damage (Wang et al., 2020; Angelova et al., 2021; Wareham et al., 2022), to which mitochondrial DNA is particularly vulnerable. The fact that Ldlr-/-.Leiden mice but not C57BL/6J mice develop mitochondrial dysfunction during aging on a chow diet advocates additional examination of the Ldlr-/-.Leiden mice as an aging model, the more so because these animals develop moderate visceral obesity and atherosclerosis on the long run, even on a normal chow diet (Verschuren et al., 2009; Gart et al., 2023). Impairment of mitochondrial function is also a key feature of the HFD-fed obese Ldlr-/-.Leiden mouse: while in the livers of the same mice as those described herein, oxidative phosphorylation and mitochondria dysfunction pathways were significantly altered by HFD feeding (Seidel et al., 2022), these pathways in the brain were not further impacted by HFD feeding. This suggests that differences seem to exist between the brain and peripheral organs such as the liver regarding the effect of additional metabolic stress from HFD feeding. It is unclear why HFD feeding does not further augment mitochondrial dysfunction in the brain. Possible explanations could be that the dysfunctionality is already maximal on chow diet and/or that the metabolic homeostasis is more tightly controlled in the brain.

In addition, we showed that HFD feeding increased neuroinflammation signaling pathway, which was in line with the changes in microglia immunophenotype that were observed in the histopathological analysis. HFD also decreased synaptogenesis signaling and SNARE signaling, which are essential pathways for synaptic vesicle exocytosis and neurotransmitter release. In addition to the impairment of synaptogenesis, diet-induced obesity has also previously been found to be associated with synaptic dysfunction and synapse loss (Bocarsly et al., 2015; Hao et al., 2016) which may be the result of synapse elimination by activated microglia (Hao et al., 2016). Interestingly, in an animal model for Alzheimer’s disease, eliminated synapses were shown to exhibit mitochondrial dysfunction (Györffy et al., 2020), a pathway that is enriched in the hippocampus of the Ldlr-/-.Leiden mouse model as described earlier.

In this study, HFD-induced neuroinflammation was accompanied by an increase in the concentration of the pro-inflammatory cytokine IL-6 in the cortex. Consistent with this, others have shown that obesity-related systemic inflammation is associated with increased systemic as well as cerebral IL-6 levels, either by local production in the brain and/or by crossing of the blood-brain barrier from the periphery (reviewed in Arnoldussen et al., 2014). The latter review suggests that increased IL-6 in the brain, especially in the hippocampus, is associated with learning and memory dysfunction through the inhibition of neurogenesis and decreased synaptic plasticity. IL-6 levels were also shown to be increased in the cortex of aged mice and were associated with diverse detrimental effects in the brain (Godbout and Johnson, 2004). Furthermore, we found a tendency towards an increase in KC concentrations in the thalamus after HFD feeding, a pro-inflammatory cytokine also known as CXCL1/2. Local production of CXCL-1 in the brain upon chronic stress stimuli has been previously described (Song et al., 2020). Although the role of CXCL1 in the brain is little known, genetic knock-out of its receptor CXCR2 has been shown to reduce neutrophil recruitment and blood-brain barrier permeability which suggest a potential role of KC in neuroinflammation (Michael et al., 2020). Altogether the HFD-induced increases in IL-6 and KC concentrations in the brain support that HFD feeding induces a pro-inflammatory milieu in line with the observed increase in neuroinflammation.

To finally test whether the aforementioned pathophysiological features can be modulated in a therapeutic setting, obese Ldlr-/-.Leiden mice were treated with an anti-C5 antibody (BB5.1), which inhibits the terminal complement pathway. Increased activity of the complement system in the brain has been observed in obesity (Graham et al., 2020) and has been shown to trigger neuroinflammatory cascades with activation of astrocytes and microglia, which may cause neurodegenerative disease (Dalakas et al., 2020). Previous studies showed that this complement system-mediated neuroinflammation can be abrogated by inhibition of MAC, the terminal complex of the complement system cascade (Fluiter et al., 2014; Michailidou et al., 2018). We previously showed in Ldlr-/-.Leiden mice that blocking C5 in the circulation with BB5.1 antibody reduced the potential for complement activation, MAC deposition and plasma concentrations of macrophage migration inhibitory factor (Seidel et al., 2022). In the present study, we found that the anti-C5 treatment did not alter neurodegeneration or astrogliosis, but partially reversed the effect of HFD on microglial immunophenotype, notably by increasing IBA-1 immunoreactivity back to chow level. In addition, on the gene expression level, the anti-C5 treatment reversed the HFD-induced downregulation of multiple pathways, including synaptogenesis. In the present study, the observed effects of the systemic anti-C5 treatment on the brain are expected to be indirect as the BB5.1 antibody and complement proteins cannot pass through intact blood-brain barrier (Alexander, 2018; Zelek et al., 2020). The anti-C5 treatment may impact microglia via indirect effects involving at least partly the C5 activation by-product C5a and MAC which do not only have inflammatory functions but can also act as important modulators of vascular inflammation and permeability. C5a can notably affect vascular inflammation by inducing adhesion molecules and several selectins (Foreman et al., 1994; Albrecht et al., 2004) and has been shown to increase blood-brain barrier permeability (Jacob and Alexander, 2014). In addition, in sublytic concentrations MAC can activate endothelial cells by modulating the secretion of pro-inflammatory mediators and by upregulating adhesion molecules (Kilgore et al., 1997). In addition, it is possible that systemic inhibition of C5 may impact microglia indirectly through the alteration of activation state of circulating immune cells or changes in circulating cytokines. However, this effect appears to be only partial, since CD68 immunoreactivity and TREM2 immunoreactivity were not affected and the neuroinflammation signalling pathway in the hippocampus was unchanged by the anti-C5 treatment. This appears not to be fully in line with previous studies showing that terminal complement pathway inhibitors induce major reduction in neuroinflammation (Fluiter et al., 2014; Michailidou et al., 2018). However, these studies were performed in the context of acute disease (i.e., experimental autoimmune encephalomyelitis, traumatic brain injury) in which the blood-brain barrier is known to be more permeable. In our study, in a context of obesity (i.e., chronic low-grade inflammation), the blood-brain barrier is likely to be less permeable and the systemic anti-C5 treatment may not have entered the brain to target local production of complement factors. In neurodegenerative diseases, reactive astrocytes have been proposed to induce the production of complement factors by reactive microglia and neurons (Stephan et al., 2012). These locally-produced complement factors are believed to subsequently label the synapses for elimination by microglia. As the systemic anti-complement C5 treatment in our study may not target this local production of complement factors, a complement-mediated elimination of synapses by microglia may explain the remaining phagocytic profile of microglia in anti C5-treated mice.

Additionally, we showed that the anti-C5 treatment did not reverse the HFD-induced increase in IL-6 or KC concentrations but increased IL-33 in the thalamus in comparison with both HFD-fed controls and chow-fed mice. Since IL-33 has been shown to have both pro- and anti-inflammatory effects in the brain (Rao et al., 2022), it is not clear whether the observed increase in IL-33 in the thalamus in this study is beneficial or deleterious. Given that the activation state of microglial cells is highly influenced by the cytokine environment (Hanisch, 2002), anti-C5 treatment-mediated changes in IL-33 may be linked to the partial reversal effect of the treatment on the expression of microglia surface markers.

## 5. Conclusion

In this study we show that Ldlr-/-.Leiden mice are more prone to develop neurodegeneration and age-related astrogliosis that is not observed in wildtype (C57BL6/J) mice. On the gene expression level, Ldlr-/-.Leiden mice exhibit pronounced mitochondrial dysfunction and impaired oxidative phosphorylation, and the pathway required for protein synthesis and repair (eIF2) is significantly inactivated in the hippocampus compared with wildtype mice. When fed an obesity-inducing HFD, Ldlr-/-.Leiden mice further exhibit microglia activation that is characterised by an immunotypic switch to a more phagocytic state, in line with what has also been reported in people with obesity or neurodegenerative disease such as Alzheimer’s disease. On the gene expression level, HFD-fed obese Ldlr-/-.Leiden also exhibit increased neuroinflammation and decreased synaptogenesis in the hippocampus. This HFD-induced pathology in Ldlr-/-.Leiden mice can also be modulated by therapeutic treatment: the microglia immunotypic switch and hippocampal gene expression is partly reversed by a systemic therapeutic antibody intervention targeting complement C5. In sum, this study provides evidence supporting the Ldlr-/-.Leiden mouse model as an appropriate model to study the development of brain pathology in the context of aging and obesity.

## Data availability statement

The transcriptomics data presented in the study are deposited in the Gene Expression Omnibus (GEO) repository (https://www.ncbi.nlm.nih.gov/gds), accession number GSE234425.

## Ethics statement

The animal study was reviewed and approved by an independent Animal Welfare Body (IVD TNO; approval numbers TNO-451 and TNO-499) under project licenses granted by the Netherlands Central Authority for Scientific Procedures on Animals (CCD; project license numbers AVD5010020172064 and AVD5010020172931).

## Author contributions

RK, KF, IM, and MM: conceptualization. FS, RK, KF, MM, and IM: methodology, writing—original draft preparation. FS, AN, NW, and IM: investigation. NG and AK: resources. FS, MC, and IM: data analysis. FS, RK, KF, NG, AK, FB, IM, and MM: data interpretation. MM and IM: supervision. MM: project administration. RK, FB, KF, and MM: funding acquisition. All authors: writing—review and editing.
