# Corrigendum: Ldlr-/-.Leiden mice develop neurodegeneration, age-dependent astrogliosis and obesity-induced changes in microglia immunophenotype which are partly reversed by complement component 5 neutralizing antibody

**DOI:** 10.3389/fncel.2023.1267913

**Published:** 2023-08-08

**Authors:** Florine Seidel, Kees Fluiter, Robert Kleemann, Nicole Worms, Anita van Nieuwkoop, Martien P. M. Caspers, Nikolaos Grigoriadis, Amanda J. Kiliaan, Frank Baas, Iliana Michailidou, Martine C. Morrison

**Affiliations:** ^1^Department of Metabolic Health Research, Netherlands Organisation for Applied Scientific Research (TNO), Leiden, Netherlands; ^2^Department of Medical Imaging, Anatomy, Preclinical Imaging Center (PRIME), Radboud Alzheimer Center, Donders Institute for Brain, Cognition, and Behavior, Radboud University Medical Center, Nijmegen, Netherlands; ^3^Department of Clinical Genetics, Leiden University Medical Center, Leiden, Netherlands; ^4^Department of Microbiology and Systems Biology, Netherlands Organisation for Applied Scientific Research (TNO), Leiden, Netherlands; ^5^Laboratory of Experimental Neurology and Neuroimmunology and the Multiple Sclerosis Center, 2^nd^ Department of Neurology, AHEPA University Hospital, Aristotle University of Thessaloniki, Thessaloniki, Greece

**Keywords:** obesity, aging, brain, neurodegeneration, astrogliosis, neuroinflammation, anti-complement component 5

In the published article there was an error. A correction has been made to the **Acknowledgments** section. This sentence previously stated:

“We would like to thank the biotechnicians from TNO Metabolic Health Research for taking excellent care of the mice used in this study.”

The corrected sentence appears below:

“We would like to thank the biotechnicians from TNO Metabolic Health Research for taking excellent care of the mice used in this study. We also thank B. P. Morgan and T. Hughes for kindly providing the BB5.1 antibody.”

The authors apologize for this error and state that this does not change the scientific conclusions of the article in any way. The original article has been updated.

