# Supplementary material for: Ldlr-/-.Leiden mice develop neurodegeneration, age-dependent astrogliosis and obesity-induced changes in microglia immunophenotype which are partly reversed by complement component 5 neutralizing antibody
Source: Front Cell Neurosci. 2023 Jun 29;17:1205261. doi: 10.3389/fncel.2023.1205261 (PMC10346859; doi:10.3389/fncel.2023.1205261)
Supplement: Supplementary file 1 [file Table_1.DOCX]

**Supplemental table 1:** **Canonical pathways analysis based on hippocampal gene expression in 50 week-old Ldlr-/-.Leiden mice vs C57BL/6J mice fed a standard chow diet.**

| Canonical pathway | -log(p-value) | z-score |
| --- | --- | --- |
| Estrogen Receptor Signaling | 24,1 | 2,9 |
| EIF2 Signaling | 23,4 | -3,9 |
| Oxidative Phosphorylation | 23,4 | -7,4 |
| Mitochondrial Dysfunction | 23,0 | 5,1 |
| mTOR Signaling | 15,5 | -0,4 |
| Regulation of eIF4 and p70S6K Signaling | 15,0 | 0,8 |
| Sirtuin Signaling Pathway | 15,0 | 2,5 |
| Huntington's Disease Signaling | 13,2 | 2,7 |
| Protein Ubiquitination Pathway | 12,6 | N/A |
| CLEAR Signaling Pathway | 12,5 | 2,8 |
| Insulin Secretion Signaling Pathway | 11,9 | 0,2 |
| Granzyme A Signaling | 11,1 | 5,8 |
| HER-2 Signaling in Breast Cancer | 10,9 | 2,6 |
| Myelination Signaling Pathway | 10,8 | 4,1 |
| NGF Signaling | 10,0 | 1,9 |
| Molecular Mechanisms of Cancer | 9,4 | N/A |
| Coronavirus Pathogenesis Pathway | 8,5 | 3,6 |
| Signaling by Rho Family GTPases | 8,2 | 3,6 |
| Synaptogenesis Signaling Pathway | 8,1 | 2,9 |
| Hypoxia Signaling in the Cardiovascular System | 7,6 | 0,3 |
| Integrin Signaling | 7,3 | 1,5 |
| Ephrin Receptor Signaling | 7,2 | 0,1 |
| Prostate Cancer Signaling | 6,8 | N/A |
| NER (Nucleotide Excision Repair, Enhanced Pathway) | 6,8 | 0,7 |
| Cardiac Hypertrophy Signaling | 6,7 | 1,8 |
| Autophagy | 6,6 | 0,7 |
| Thrombin Signaling | 6,5 | 2,4 |
| Germ Cell-Sertoli Cell Junction Signaling | 6,2 | N/A |
| RANK Signaling in Osteoclasts | 6,2 | 2,5 |
| Superpathway of Inositol Phosphate Compounds | 6,1 | 2,6 |
| Epithelial Adherens Junction Signaling | 6,0 | -1,1 |
| Androgen Signaling | 6,0 | 3,1 |
| Macropinocytosis Signaling | 5,9 | 1,2 |
| Virus Entry via Endocytic Pathways | 5,9 | N/A |
| 3-phosphoinositide Biosynthesis | 5,9 | 1,9 |
| RAC Signaling | 5,8 | 0,9 |
| BAG2 Signaling Pathway | 5,7 | -1,5 |
| Acute Myeloid Leukemia Signaling | 5,7 | 1,7 |
| RHOGDI Signaling | 5,6 | -1,7 |
| Role of NFAT in Cardiac Hypertrophy | 5,6 | 2,9 |
| Chronic Myeloid Leukemia Signaling | 5,5 | 2,4 |
| MicroRNA Biogenesis Signaling Pathway | 5,4 | -0,8 |
| Apelin Endothelial Signaling Pathway | 5,4 | 2,2 |
| fMLP Signaling in Neutrophils | 5,3 | 0,3 |
| Adipogenesis pathway | 5,2 | 1,0 |
| AMPK Signaling | 5,1 | 1,3 |
| Glioma Signaling | 5,1 | 2,5 |
| P2Y Purigenic Receptor Signaling Pathway | 5,1 | 1,7 |
| GNRH Signaling | 5,1 | 2,3 |
| 3-phosphoinositide Degradation | 5,1 | 1,5 |
| Glucocorticoid Receptor Signaling | 5,0 | N/A |
| Renal Cell Carcinoma Signaling | 5,0 | 0,7 |
| Actin Cytoskeleton Signaling | 5,0 | 1,2 |
| HIF1Î± Signaling | 5,0 | 2,1 |
| Axonal Guidance Signaling | 4,9 | N/A |
| Hereditary Breast Cancer Signaling | 4,9 | N/A |
| RHOA Signaling | 4,8 | 0,6 |
| Xenobiotic Metabolism General Signaling Pathway | 4,8 | 3,2 |
| Pulmonary Fibrosis Idiopathic Signaling Pathway | 4,8 | 2,3 |
| HGF Signaling | 4,8 | 2,7 |
| CNTF Signaling | 4,7 | 0,8 |
| Aldosterone Signaling in Epithelial Cells | 4,7 | 3,1 |
| PI3K/AKT Signaling | 4,7 | -1,5 |
| CCR3 Signaling in Eosinophils | 4,6 | 2,3 |
| RAR Activation | 4,6 | N/A |
| Reelin Signaling in Neurons | 4,6 | 2,5 |
| Endocannabinoid Developing Neuron Pathway | 4,5 | 0,5 |
| 14-3-3-mediated Signaling | 4,5 | 0,8 |
| Phagosome Maturation | 4,5 | N/A |
| WNT/Î²-catenin Signaling | 4,5 | 0,0 |
| Neutrophil Extracellular Trap Signaling Pathway | 4,5 | -2,9 |
| Unfolded protein response | 4,5 | -0,7 |
| D-myo-inositol-5-phosphate Metabolism | 4,4 | 1,0 |
| Production of Nitric Oxide and Reactive Oxygen Species in Macrophages | 4,4 | 2,7 |
| D-myo-inositol (1,4,5,6)-Tetrakisphosphate Biosynthesis | 4,4 | 0,7 |
| D-myo-inositol (3,4,5,6)-tetrakisphosphate Biosynthesis | 4,4 | 0,7 |
| Melanocyte Development and Pigmentation Signaling | 4,4 | 0,9 |
| Ceramide Signaling | 4,4 | 0,0 |
| CSDE1 Signaling Pathway | 4,4 | -1,0 |
| PEDF Signaling | 4,4 | 1,7 |
| Chemokine Signaling | 4,3 | 1,5 |
| Assembly of RNA Polymerase II Complex | 4,3 | -1,7 |
| Paxillin Signaling | 4,3 | 1,4 |
| LPS-stimulated MAPK Signaling | 4,3 | 1,5 |
| Small Cell Lung Cancer Signaling | 4,3 | 1,6 |
| Hepatic Fibrosis Signaling Pathway | 4,2 | 1,4 |
| IL-8 Signaling | 4,2 | 1,7 |
| GÎ±q Signaling | 4,2 | 2,7 |
| Mouse Embryonic Stem Cell Pluripotency | 4,2 | 1,5 |
| ERK/MAPK Signaling | 4,2 | 1,4 |
| Senescence Pathway | 4,2 | 0,8 |
| Telomerase Signaling | 4,2 | -0,8 |
| ERBB4 Signaling | 4,1 | 1,5 |
| PTEN Signaling | 4,1 | -0,8 |
| HOTAIR Regulatory Pathway | 4,1 | 2,1 |
| MSP-RON Signaling In Cancer Cells Pathway | 4,1 | 1,2 |
| CXCR4 Signaling | 4,1 | 0,8 |
| PDGF Signaling | 4,0 | 2,0 |
| ILK Signaling | 4,0 | 0,8 |
| Protein Kinase A Signaling | 4,0 | 0,2 |
| VEGF Signaling | 3,9 | 1,0 |
| Spliceosomal Cycle | 3,9 | -1,1 |
| FAT10 Signaling Pathway | 3,9 | -1,0 |
| IL-1 Signaling | 3,9 | 2,7 |
| ERK5 Signaling | 3,8 | 0,0 |
| Apoptosis Signaling | 3,8 | -0,2 |
| NF-ÎºB Activation by Viruses | 3,8 | 2,1 |
| Synaptic Long Term Potentiation | 3,7 | 2,2 |
| Insulin Receptor Signaling | 3,7 | 0,3 |
| CD27 Signaling in Lymphocytes | 3,7 | 2,4 |
| Sertoli Cell-Sertoli Cell Junction Signaling | 3,7 | N/A |
| UVA-Induced MAPK Signaling | 3,7 | 0,2 |
| Ephrin B Signaling | 3,7 | 1,5 |
| Adrenomedullin signaling pathway | 3,6 | 1,8 |
| Ovarian Cancer Signaling | 3,6 | 1,5 |
| Regulation of the Epithelial-Mesenchymal Transition Pathway | 3,6 | N/A |
| NRF2-mediated Oxidative Stress Response | 3,6 | 1,4 |
| Semaphorin Neuronal Repulsive Signaling Pathway | 3,6 | -0,1 |
| Sumoylation Pathway | 3,6 | -0,2 |
| Glioblastoma Multiforme Signaling | 3,5 | 1,2 |
| Nucleotide Excision Repair Pathway | 3,5 | N/A |
| Xenobiotic Metabolism Signaling | 3,5 | N/A |
| SNARE Signaling Pathway | 3,4 | 0,8 |
| Actin Nucleation by ARP-WASP Complex | 3,4 | -1,8 |
| FLT3 Signaling in Hematopoietic Progenitor Cells | 3,4 | 0,6 |
| Î±-Adrenergic Signaling | 3,4 | 1,7 |
| Endometrial Cancer Signaling | 3,4 | 1,2 |
| Remodeling of Epithelial Adherens Junctions | 3,3 | -2,5 |
| Melanoma Signaling | 3,3 | 0,9 |
| Cardiac Hypertrophy Signaling (Enhanced) | 3,2 | 2,9 |
| ID1 Signaling Pathway | 3,2 | 2,1 |
| PPAR Signaling | 3,2 | -0,5 |
| Induction of Apoptosis by HIV1 | 3,2 | 0,2 |
| Corticotropin Releasing Hormone Signaling | 3,2 | 1,7 |
| UVC-Induced MAPK Signaling | 3,1 | 1,1 |
| Role of Tissue Factor in Cancer | 3,1 | N/A |
| Estrogen-Dependent Breast Cancer Signaling | 3,1 | 1,0 |
| Type II Diabetes Mellitus Signaling | 3,1 | 1,6 |
| Circadian Rhythm Signaling | 3,1 | N/A |
| PPARÎ±/RXRÎ± Activation | 3,1 | 1,2 |
| Clathrin-mediated Endocytosis Signaling | 3,1 | N/A |
| IGF-1 Signaling | 3,0 | 1,3 |
| Neuregulin Signaling | 3,0 | 1,6 |
| Neurotrophin/TRK Signaling | 3,0 | 1,4 |
| UVB-Induced MAPK Signaling | 3,0 | 2,1 |
| Notch Signaling | 3,0 | -1,0 |
| JAK/STAT Signaling | 3,0 | 2,0 |
| Mitotic Roles of Polo-Like Kinase | 3,0 | 0,0 |
| Pancreatic Adenocarcinoma Signaling | 3,0 | 1,8 |
| Colorectal Cancer Metastasis Signaling | 2,9 | 2,0 |
| Gap Junction Signaling | 2,9 | N/A |
| Regulation Of The Epithelial Mesenchymal Transition In Development Pathway | 2,9 | 1,0 |
| Necroptosis Signaling Pathway | 2,9 | -1,2 |
| TNFR2 Signaling | 2,9 | 1,9 |
| Regulation of Actin-based Motility by Rho | 2,9 | -0,4 |
| Oxytocin Signaling Pathway | 2,8 | 2,6 |
| TR/RXR Activation | 2,8 | N/A |
| Ribonucleotide Reductase Signaling Pathway | 2,8 | 1,7 |
| Inhibition of ARE-Mediated mRNA Degradation Pathway | 2,8 | -1,3 |
| G Beta Gamma Signaling | 2,8 | 2,0 |
| Renin-Angiotensin Signaling | 2,7 | 2,7 |
| Opioid Signaling Pathway | 2,7 | 2,8 |
| RAN Signaling | 2,7 | -2,3 |
| Natural Killer Cell Signaling | 2,7 | 2,7 |
| Endocannabinoid Cancer Inhibition Pathway | 2,7 | -0,9 |
| TWEAK Signaling | 2,7 | 0,5 |
| BEX2 Signaling Pathway | 2,7 | 1,4 |
| FGF Signaling | 2,6 | 1,7 |
| p53 Signaling | 2,6 | -0,4 |
| FcÎ³ Receptor-mediated Phagocytosis in Macrophages and Monocytes | 2,6 | 1,1 |
| Non-Small Cell Lung Cancer Signaling | 2,6 | 0,9 |
| Role Of Osteoclasts In Rheumatoid Arthritis Signaling Pathway | 2,6 | 1,4 |
| Dopamine-DARPP32 Feedback in cAMP Signaling | 2,6 | 2,5 |
| Thrombopoietin Signaling | 2,6 | 1,5 |
| Cholecystokinin/Gastrin-mediated Signaling | 2,6 | 1,0 |
| Thyroid Cancer Signaling | 2,6 | 2,4 |
| Prolactin Signaling | 2,6 | 2,0 |
| Ferroptosis Signaling Pathway | 2,6 | 0,6 |
| Superpathway of Geranylgeranyldiphosphate Biosynthesis I (via Mevalonate) | 2,5 | 0,3 |
| GDNF Family Ligand-Receptor Interactions | 2,5 | 1,3 |
| PAK Signaling | 2,5 | 1,7 |
| Endothelin-1 Signaling | 2,4 | 2,4 |
| NAD Signaling Pathway | 2,4 | 1,3 |
| ERBB Signaling | 2,4 | 1,9 |
| Antiproliferative Role of Somatostatin Receptor 2 | 2,4 | 0,5 |
| Cancer Drug Resistance By Drug Efflux | 2,4 | 0,2 |
| Î³-linolenate Biosynthesis II (Animals) | 2,3 | 1,4 |
| TNFR1 Signaling | 2,3 | 0,7 |
| Role of Osteoblasts, Osteoclasts and Chondrocytes in Rheumatoid Arthritis | 2,3 | N/A |
| Lymphotoxin Î² Receptor Signaling | 2,3 | 1,1 |
| Apelin Cardiomyocyte Signaling Pathway | 2,3 | 1,8 |
| PFKFB4 Signaling Pathway | 2,2 | 0,8 |
| Death Receptor Signaling | 2,2 | 0,8 |
| EGF Signaling | 2,2 | 2,1 |
| TCA Cycle II (Eukaryotic) | 2,2 | 1,3 |
| Mechanisms of Viral Exit from Host Cells | 2,2 | N/A |
| STAT3 Pathway | 2,1 | 2,0 |
| Regulation Of The Epithelial Mesenchymal Transition By Growth Factors Pathway | 2,1 | 2,1 |
| Role of MAPK Signaling in Promoting the Pathogenesis of Influenza | 2,1 | -0,2 |
| Tight Junction Signaling | 2,1 | N/A |
| Cyclins and Cell Cycle Regulation | 2,1 | -0,5 |
| ERB2-ERBB3 Signaling | 2,1 | 1,1 |
| CDK5 Signaling | 2,1 | 1,5 |
| p38 MAPK Signaling | 2,0 | 0,4 |
| Sphingosine-1-phosphate Signaling | 2,0 | 1,6 |
|  |  |  |

Only most significantly enriched canonical pathways are displayed (p ≤ 0.01). The z-score indicates the predicted activation of a canonical pathway: z-score ≤ -2 indicates relevant inhibition of the pathway (shown in dark blue); z-score ≥ 2 indicates relevant activation of the pathway (shown in dark orange).

**Supplemental table 2: Canonical pathways analysis based on hippocampal gene expression in 50 week-old HFD-fed Ldlr-/-.Leiden mice vs 50 week-old chow-fed Ldlr-/-.Leiden mice.**

| Canonical pathway | -log(p-value) | z-score |
| --- | --- | --- |
| Synaptogenesis Signaling Pathway | 5,4 | -2,1 |
| Superpathway of Cholesterol Biosynthesis | 5,1 | -2,3 |
| Coronavirus Pathogenesis Pathway | 4,9 | -2,7 |
| Calcium Signaling | 4,8 | -0,5 |
| G-Protein Coupled Receptor Signaling | 3,8 | -1,3 |
| SNARE Signaling Pathway | 3,8 | -2,8 |
| Dilated Cardiomyopathy Signaling Pathway | 3,7 | -1,1 |
| Oxytocin Signaling Pathway | 3,7 | -2,0 |
| Corticotropin Releasing Hormone Signaling | 3,7 | -1,7 |
| Cholesterol Biosynthesis I | 3,5 | -2,2 |
| Cholesterol Biosynthesis II (via 24,25-dihydrolanosterol) | 3,5 | -2,2 |
| Cholesterol Biosynthesis III (via Desmosterol) | 3,5 | -2,2 |
| Insulin Secretion Signaling Pathway | 3,2 | -0,2 |
| IL-10 Signaling | 3,1 | -0,9 |
| Netrin Signaling | 3,1 | 0,6 |
| TNFR2 Signaling | 3,1 | -0,4 |
| Role of NFAT in Cardiac Hypertrophy | 3,0 | -1,5 |
| Acetate Conversion to Acetyl-CoA | 2,9 | N/A |
| GNRH Signaling | 2,8 | -2,8 |
| Superpathway of Geranylgeranyldiphosphate Biosynthesis I (via Mevalonate) | 2,8 | -1,3 |
| Pathogen Induced Cytokine Storm Signaling Pathway | 2,7 | 1,4 |
| TWEAK Signaling | 2,7 | -0,4 |
| IGF-1 Signaling | 2,6 | -0,7 |
| Neuroinflammation Signaling Pathway | 2,6 | 2,6 |
| Cardiac Hypertrophy Signaling | 2,5 | -2,1 |
| Role of PKR in Interferon Induction and Antiviral Response | 2,5 | 0,3 |
| IL-15 Production | 2,5 | 0,5 |
| Cellular Effects of Sildenafil (Viagra) | 2,4 | N/A |
| Cardiac Hypertrophy Signaling (Enhanced) | 2,4 | -1,8 |
| Role Of Osteoclasts In Rheumatoid Arthritis Signaling Pathway | 2,4 | -0,2 |
| Mevalonate Pathway I | 2,4 | -1,0 |
| Circadian Rhythm Signaling | 2,4 | N/A |
| April Mediated Signaling | 2,3 | 0,0 |
| B Cell Activating Factor Signaling | 2,3 | 0,0 |
| EIF2 Signaling | 2,3 | 2,1 |
| Protein Kinase A Signaling | 2,3 | -0,2 |
| Acute Phase Response Signaling | 2,3 | 1,2 |
| Crosstalk between Dendritic Cells and Natural Killer Cells | 2,2 | 1,0 |
| Inositol Pyrophosphates Biosynthesis | 2,2 | N/A |
| CD40 Signaling | 2,2 | -0,7 |
| Phagosome Formation | 2,2 | -0,3 |
| CD27 Signaling in Lymphocytes | 2,1 | -1,9 |
| RHOGDI Signaling | 2,1 | 2,0 |
| Renin-Angiotensin Signaling | 2,1 | -2,3 |
| Myelination Signaling Pathway | 2,1 | -1,3 |
| cAMP-mediated signaling | 2,1 | -0,4 |
| Semaphorin Neuronal Repulsive Signaling Pathway | 2,1 | -0,8 |
| Ephrin A Signaling | 2,1 | N/A |
| Death Receptor Signaling | 2,1 | 0,3 |
| LXR/RXR Activation | 2,1 | 0,7 |
| TR/RXR Activation | 2,0 | N/A |
| White Adipose Tissue Browning Pathway | 2,0 | -1,7 |
| Role of Macrophages, Fibroblasts and Endothelial Cells in Rheumatoid Arthritis | 2,0 | N/A |
| Axonal Guidance Signaling | 2,0 | N/A |
|  |  |  |

Only most significantly enriched canonical pathways are displayed (p ≤ 0.01). The z-score indicates the predicted activation of a canonical pathway: z-score ≤ -2 indicates relevant inhibition of the pathway (shown in dark blue); z-score ≥ 2 indicates relevant activation of the pathway (shown in dark orange).

**Supplemental table 3: Canonical pathways analysis based on hippocampal gene expression in 50 week-old HFD-fed Ldlr-/-.Leiden + BB5.1 mice vs 50 week-old HFD-fed Ldlr-/-.Leiden mice.**

| Canonical pathway | -log(p-value) | z-score |
| --- | --- | --- |
| Calcium Signaling | 6,8 | 1,1 |
| Synaptogenesis Signaling Pathway | 6,5 | 3,8 |
| SNARE Signaling Pathway | 6,4 | 3,8 |
| Hepatic Fibrosis Signaling Pathway | 6,1 | -0,2 |
| GNRH Signaling | 5,9 | 3,1 |
| IL-17A Signaling in Gastric Cells | 5,8 | 1,6 |
| Myelination Signaling Pathway | 5,7 | 0,7 |
| Dilated Cardiomyopathy Signaling Pathway | 5,7 | 1,0 |
| 4-1BB Signaling in T Lymphocytes | 5,6 | 1,0 |
| Cardiac Hypertrophy Signaling | 5,2 | 2,0 |
| Role of NFAT in Cardiac Hypertrophy | 5,1 | 2,4 |
| LPS-stimulated MAPK Signaling | 5,0 | 1,9 |
| Paxillin Signaling | 5,0 | 1,8 |
| Cardiac Hypertrophy Signaling (Enhanced) | 5,0 | 1,4 |
| Insulin Secretion Signaling Pathway | 4,8 | 2,1 |
| ATM Signaling | 4,8 | 0,8 |
| Molecular Mechanisms of Cancer | 4,7 | N/A |
| BMP signaling pathway | 4,7 | 1,9 |
| Endocannabinoid Neuronal Synapse Pathway | 4,6 | 1,1 |
| G-Protein Coupled Receptor Signaling | 4,6 | 2,9 |
| ILK Signaling | 4,6 | 1,7 |
| Netrin Signaling | 4,6 | -0,3 |
| AMPK Signaling | 4,5 | 1,5 |
| Corticotropin Releasing Hormone Signaling | 4,5 | 2,2 |
| Pulmonary Fibrosis Idiopathic Signaling Pathway | 4,5 | 1,4 |
| Autophagy | 4,5 | 0,8 |
| IL-17A Signaling in Fibroblasts | 4,3 | N/A |
| Renin-Angiotensin Signaling | 4,3 | 2,3 |
| Role Of Osteoclasts In Rheumatoid Arthritis Signaling Pathway | 4,2 | 1,7 |
| Oxytocin Signaling Pathway | 4,1 | 2,4 |
| Role of Osteoblasts, Osteoclasts and Chondrocytes in Rheumatoid Arthritis | 4,1 | N/A |
| Agrin Interactions at Neuromuscular Junction | 4,1 | 1,5 |
| RANK Signaling in Osteoclasts | 4,1 | 1,2 |
| PCP (Planar Cell Polarity) Pathway | 4,0 | 1,3 |
| Coronavirus Pathogenesis Pathway | 4,0 | 2,3 |
| IL-10 Signaling | 3,9 | 0,7 |
| April Mediated Signaling | 3,9 | 0,0 |
| B Cell Activating Factor Signaling | 3,8 | 0,0 |
| Ribonucleotide Reductase Signaling Pathway | 3,8 | 1,8 |
| Acute Phase Response Signaling | 3,8 | -0,5 |
| Activation of IRF by Cytosolic Pattern Recognition Receptors | 3,7 | 0,9 |
| Mitochondrial Dysfunction | 3,7 | 2,5 |
| Role of Macrophages, Fibroblasts and Endothelial Cells in Rheumatoid Arthritis | 3,6 | N/A |
| IL-17A Signaling in Airway Cells | 3,6 | 1,0 |
| Role of IL-17A in Arthritis | 3,5 | N/A |
| CDK5 Signaling | 3,5 | 0,0 |
| Role of CHK Proteins in Cell Cycle Checkpoint Control | 3,5 | -0,4 |
| Cancer Drug Resistance By Drug Efflux | 3,5 | -1,0 |
| Chemokine Signaling | 3,4 | 2,1 |
| Circadian Rhythm Signaling | 3,4 | N/A |
| Germ Cell-Sertoli Cell Junction Signaling | 3,4 | N/A |
| BEX2 Signaling Pathway | 3,4 | -1,3 |
| Senescence Pathway | 3,4 | -1,7 |
| Synaptic Long Term Potentiation | 3,3 | 2,8 |
| p38 MAPK Signaling | 3,3 | 1,3 |
| IL-1 Signaling | 3,3 | 0,6 |
| UVC-Induced MAPK Signaling | 3,3 | 0,7 |
| WNT/Î²-catenin Signaling | 3,2 | 0,7 |
| Axonal Guidance Signaling | 3,2 | N/A |
| Breast Cancer Regulation by Stathmin1 | 3,2 | 0,6 |
| IL-22 Signaling | 3,2 | 1,3 |
| Chronic Myeloid Leukemia Signaling | 3,1 | 0,0 |
| Sertoli Cell-Sertoli Cell Junction Signaling | 3,1 | N/A |
| PTEN Signaling | 3,1 | 1,8 |
| Tight Junction Signaling | 3,1 | N/A |
| Angiopoietin Signaling | 3,1 | 1,0 |
| GDNF Family Ligand-Receptor Interactions | 3,1 | 0,9 |
| Signaling by Rho Family GTPases | 3,1 | 1,5 |
| Inhibition of Angiogenesis by TSP1 | 3,1 | 0,8 |
| S100 Family Signaling Pathway | 3,0 | 2,4 |
| Role of MAPK Signaling in Promoting the Pathogenesis of Influenza | 3,0 | 1,9 |
| Endocannabinoid Developing Neuron Pathway | 3,0 | 2,3 |
| Role Of Chondrocytes In Rheumatoid Arthritis Signaling Pathway | 3,0 | 0,5 |
| IL-7 Signaling Pathway | 3,0 | 1,6 |
| Integrin Signaling | 3,0 | 0,7 |
| Sumoylation Pathway | 3,0 | 1,5 |
| Protein Kinase A Signaling | 3,0 | -0,2 |
| CD40 Signaling | 3,0 | 0,3 |
| Neuroinflammation Signaling Pathway | 3,0 | 0,2 |
| G Beta Gamma Signaling | 3,0 | 0,8 |
| Role of BRCA1 in DNA Damage Response | 2,9 | 0,0 |
| ERBB Signaling | 2,9 | 2,1 |
| GÎ±12/13 Signaling | 2,8 | 1,3 |
| PFKFB4 Signaling Pathway | 2,8 | 2,1 |
| CREB Signaling in Neurons | 2,7 | 2,3 |
| GADD45 Signaling | 2,7 | 1,7 |
| Role of PKR in Interferon Induction and Antiviral Response | 2,7 | 0,8 |
| Role of MAPK Signaling in the Pathogenesis of Influenza | 2,7 | N/A |
| tRNA Charging | 2,7 | 1,1 |
| UVA-Induced MAPK Signaling | 2,7 | 0,4 |
| White Adipose Tissue Browning Pathway | 2,7 | 1,6 |
| Thrombin Signaling | 2,6 | 1,8 |
| Regulation of eIF4 and p70S6K Signaling | 2,6 | 0,0 |
| Colorectal Cancer Metastasis Signaling | 2,6 | 1,0 |
| Factors Promoting Cardiogenesis in Vertebrates | 2,6 | 2,5 |
| UDP-N-acetyl-D-galactosamine Biosynthesis II | 2,6 | N/A |
| Synaptic Long Term Depression | 2,6 | 1,2 |
| Prostate Cancer Signaling | 2,6 | N/A |
| Neuropathic Pain Signaling In Dorsal Horn Neurons | 2,6 | 3,5 |
| UVB-Induced MAPK Signaling | 2,6 | 1,1 |
| Xenobiotic Metabolism Signaling | 2,5 | N/A |
| Hereditary Breast Cancer Signaling | 2,5 | N/A |
| IL-6 Signaling | 2,5 | 0,3 |
| Dopamine-DARPP32 Feedback in cAMP Signaling | 2,5 | 2,1 |
| Induction of Apoptosis by HIV1 | 2,5 | 1,0 |
| Opioid Signaling Pathway | 2,4 | 1,2 |
| Estrogen Receptor Signaling | 2,4 | 1,2 |
| Role of MAPK Signaling in Inhibiting the Pathogenesis of Influenza | 2,4 | 1,9 |
| RAR Activation | 2,4 | N/A |
| Cholecystokinin/Gastrin-mediated Signaling | 2,4 | 1,2 |
| 3-phosphoinositide Degradation | 2,4 | 1,8 |
| EGF Signaling | 2,3 | 0,4 |
| Cellular Effects of Sildenafil (Viagra) | 2,3 | N/A |
| JAK/STAT Signaling | 2,3 | -1,3 |
| Apelin Pancreas Signaling Pathway | 2,3 | -1,1 |
| Role of OCT4 in Mammalian Embryonic Stem Cell Pluripotency | 2,3 | N/A |
| CDP-diacylglycerol Biosynthesis I | 2,3 | 1,3 |
| Parkinson's Signaling | 2,2 | N/A |
| Type II Diabetes Mellitus Signaling | 2,2 | 1,0 |
| CXCR4 Signaling | 2,2 | 1,3 |
| PEDF Signaling | 2,2 | -0,3 |
| Glioma Signaling | 2,2 | 1,4 |
| Ephrin A Signaling | 2,2 | N/A |
| iNOS Signaling | 2,2 | -0,8 |
| Pancreatic Adenocarcinoma Signaling | 2,2 | 0,6 |
| PI3K/AKT Signaling | 2,2 | -2,9 |
| Neurovascular Coupling Signaling Pathway | 2,2 | 2,2 |
| IL-33 Signaling Pathway | 2,2 | 1,3 |
| Glioblastoma Multiforme Signaling | 2,2 | 0,8 |
| Folate Transformations I | 2,2 | N/A |
| FGF Signaling | 2,1 | 2,3 |
| Ovarian Cancer Signaling | 2,1 | 1,3 |
| Phosphatidylglycerol Biosynthesis II (Non-plastidic) | 2,1 | 1,3 |
| Melanoma Signaling | 2,1 | 0,8 |
| CLEAR Signaling Pathway | 2,1 | 2,1 |
| PAK Signaling | 2,0 | 2,1 |
| HGF Signaling | 2,0 | 1,7 |
| TNFR1 Signaling | 2,0 | 0,4 |
| Amyloid Processing | 2,0 | N/A |
|  |  |  |

Only most significantly enriched canonical pathways are displayed (p ≤ 0.01). The z-score indicates the predicted activation of a canonical pathway: z-score ≤ -2 indicates relevant inhibition of the pathway (shown in dark blue); z-score ≥ 2 indicates relevant activation of the pathway (shown in dark orange).
